# Development of a Film-Forming Wound Dressing from *Periplaneta americana* Grease: Formulation, Characterization, and Bioevaluation

**DOI:** 10.3390/ph19030401

**Published:** 2026-02-28

**Authors:** Qian Wang, Zhuohui He, Siyu Ji, Jie Zhao, Pengfei Gao, Yunchuan Yang, Lijuan Li, Hairong Zhao, Chenggui Zhang

**Affiliations:** 1 College of Pharmacy, Dali University, Dali 671000, China; 2 Yunnan Provincial Key Laboratory of Entomological Biopharmaceutical R&D, Dali University, Dali 671000, China; 3 National-Local Joint Engineering Research Center of Entomoceutics, Dali University, Dali 671000, China; 4 The First Affiliated Hospital of Dali University, Dali 671000, China

**Keywords:** *Periplaneta americana* grease, film-forming agent, wound healing, antioxidant activity

## Abstract

**Background**: *Periplaneta americana* grease (PAG), a lipid-rich fraction with documented wound-repair properties, remains challenging. This study aimed to develop a stable and patient-friendly film-forming agent (PAP) from PAG for topical wound management. **Methods**: The chemical profile of PAG was characterized with GC-MS. The formulation was optimized via single-factor and orthogonal experimental design. Comprehensive physicochemical characterization was performed. A vehicle control (film without PAG) was used to isolate PAG’s bioactive effects. In vitro, antioxidant (DPPH/ABTS assays) and antibacterial activity were evaluated. In vivo efficacy was assessed using a murine full-thickness wound model (mice, 150 µL applied 3 times daily for 10 days), with bFGF and Kangfuxin solution as positive controls. Histological analysis was conducted on healed tissue. **Results**: GC-MS revealed PAG’s complex composition, rich in sterols, terpenoids, and heterocyclic compounds. The optimized PAP formed a uniform, flexible film with suitable mechanical strength and shear-thinning rheology. PAP showed significant antioxidant activity and selective antibacterial activity against *Staphylococcus aureus*. In the wound model, PAP treatment significantly accelerated wound closure, achieving a 98.2% healing rate by day 10, comparable to positive controls and significantly superior to the vehicle control. Histology demonstrated enhanced re-epithelialization, reduced inflammation, and improved collagen organization. **Conclusions**: PAP was successfully formulated into a multifunctional film-forming agent that addresses key barriers to healing—infection, oxidative stress, and tissue regeneration. The results demonstrate its potential as an innovative therapeutic strategy for wound care.

## 1. Introduction

The *Periplaneta americana* L. (*P. americana* L.), widely known as the cockroach, has a long history of use in traditional Chinese medicine due to its recognized medicinal properties [1,2]. It is documented in ancient texts such as the Compendium of Materia Medica for its ability to “disperse stasis and soften hard masses, dispel cold and expel heat, promote qi circulation and activate meridians [3]”. Additionally, folk practices have utilized dried insect bodies to promote wound healing [4,5,6].

With advances in modern pharmaceutical sciences, the therapeutic potential of *P. americana* L. has been further validated. Several formulations derived from *P. americana* L. are now clinically established, including Kangfuxin Liquid (KFX) [7], Xinmailong Injection [8], Ganlong Capsule [9], and Xiaozheng Yigan Tablet [10]. KFX, in particular, has been used for over two decades and is known to enhance epidermal cell proliferation, granulation tissue formation, and tissue repair, making it a common choice for various cutaneous ulcers and other wound types [11]. Other preparations, such as sprays and oral extracts, are also utilized for the management of burns, scalds, and lacerations [12]. Our previous work demonstrated that e *P. americana* L. extract facilitates recovery in a dextran sodium sulfate-induced murine model of ulcerative colitis [13]. Furthermore, studies have indicated that the grease component of *P. americana* L. extract exhibits notable wound-repairing effects [14,15,16].

The grease fraction, herein referred to as *P. americana* L. grease (PAG), is obtained from the upper layer of a water–ethanol co-extraction [17,18]. Historically regarded as a by-product and often discarded. However, recent evidence suggests that PAG possesses significant bioactivity, including tissue-repair promotion and antioxidant properties [19,20,21]. Importantly, PAG shows no obvious acute toxicity [18,22] biological applications.

While existing *P. americana* L.-based formulations like KFX are therapeutically effective, conventional liquid or semi-solid dosage forms present several limitations [19]. These include poor adhesion to moist or mobile wound surfaces, lack of sustained drug release, frequent dosing requirements, and potential messiness during application—all of which may compromise patient compliance and treatment outcomes. An ideal wound therapy should not only facilitate tissue regeneration but also address critical pathophysiological barriers to healing, such as microbial infection and excessive oxidative stress at the wound site [23]. Chronic wounds, in particular, are often characterized by high bacterial load and elevated reactive oxygen species (ROS), which sustain inflammation, damage cells, and impede repair [12,24]. Therefore, a multifunctional wound care agent combining antimicrobial and antioxidant activities and pro-healing activities is highly desirable for comprehensive wound management [25]. In this context, film-forming systems present a promising alternative. Upon application, these formulations rapidly dry to form a transparent, flexible, and adherent polymeric film that conforms to wound contours. This film acts as a physical barrier against contaminants, maintains a moist healing environment, and localizes active ingredients at the wound site—thereby enhancing efficacy and reducing systemic exposure. Moreover, such systems can be designed to modulate drug release, potentially decreasing application frequency and improving patient adherence.

Building on the established pro-healing activity and favorable safety profile of PAG, we hypothesized that developing PAG into a novel film-forming coating agent could synergistically address the aforementioned limitations of conventional dosage forms. Preliminary studies suggest that PAG itself exhibits intrinsic antimicrobial and antioxidant activities, which are crucial yet underexploited assets for comprehensive wound care. Therefore, this study aimed to valorize this bioactive fraction through innovative formulation. Our specific objectives were to: (1) develop and optimize a stable film-forming coating agent based on PAG; (2) characterize its physical properties and in vitro biological activities, with a focus on antimicrobial and antioxidant potential; and (3) conduct a preliminary efficacy evaluation in a relevant wound model. The overarching goal is to advance a multifunctional, patient-friendly therapeutic candidate for wound management and to establish a foundation for its further pharmaceutical development.

## 2. Results

### 2.1. Chemical Composition of PAG

A comprehensive GC-MS-based metabolomic analysis of PAG led to the identification of a diverse array of chemical constituents (Table 1). The metabolome was predominantly composed of heterocyclic compounds (e.g., 2,3,5-trimethylpyrrole, 9.77%), alkanolamines, hydrocarbons, and terpenoids. Notable bioactive classes included alcohols and amines, such as O-pentylhydroxylamine (7.21%), and sterol-like analogs including levo-borneol and terpenoids such as β-pinene. This complex mixture of heterocycles, alkanolamines, and sterol-like compounds is consistent with reported bioactive fractions of *P. americana* and provides a chemical basis for its observed biological activities. While the relative abundance of specific compounds may vary with insect age, diet, and habitat—a common characteristic of natural products—the overarching classes of bioactive components (e.g., sterols, terpenoids, fatty acid derivatives) identified herein are likely to be conserved, supporting the reproducibility of their functional effects. Furthermore, we hypothesize that the wound-healing efficacy may be attributed to a synergistic action of these components, where sterols and terpenoids contribute to anti-inflammatory and cell-proliferative activities, while fatty acid derivatives may enhance membrane fluidity and repair.

### 2.2. Formulation Optimization of the Film-Forming Agent

#### 2.2.1. Optimal Concentration Ranges of Key Components Identified by Single-Factor Screening

The influence of six critical components on film quality was systematically evaluated using a composite scoring system (Table 2). The composite score for each trial was calculated as the sum of its scores across five parameters: film formation time, film integrity, homogeneity, bubble formation, and flexibility. The results from these single-factor experiments are summarized across multiple tables and identify the optimal concentration range for each component:

Polymer Matrix: The concentration of the primary film-forming polymer, PVA-124, was critical. Amounts below 4.0 g resulted in poorly formed, fragile films, while exceeding 8.0 g led to excessive viscosity and rigidity. A dosage between 4.0 and 6.0 g yielded the highest composite score (Table 2), balancing good film-forming ability with suitable flexibility.

Secondary Polymer & Plasticizer: The addition of PVP (0.4–0.6 g) enhanced film toughness and adhesion (Table 3). Glycerol, as a plasticizer, was optimal between 1.0 and 2.0 g, providing adequate moisturization and film plasticity without causing excessive flowability (Table 4).

Solvent & Emulsifier: Anhydrous ethanol (4–6 mL) facilitated rapid film formation and acted as a penetration enhancer, with its concentration finely tuned to balance evaporation rate and solution viscosity (Table 5). Tween-80, used as an emulsifier to homogenize the PAG within the aqueous phase, performed best at 0.05–0.15 g, ensuring a stable emulsion without adversely affecting film quality (Table 6).

Active Ingredient: The loading of the bioactive component, PAG, was optimized to ensure efficacy while maintaining physical stability. A concentration between 0.2 and 0.4 g prevented issues like phase separation or precipitation and was selected for further development (Table 7).

Therefore, the optimal ranges determined were: PVA-124, 4.0–6.0 g; PVP, 0.4–0.6 g; glycerol, 1.0–2.0 g; anhydrous ethanol, 4–6 mL; Tween-80, 0.05–0.15 g; and PAG, 0.2–0.4 g.

#### 2.2.2. Orthogonal Design Yields the Optimal Formulation Ratio

**Figure 1:**
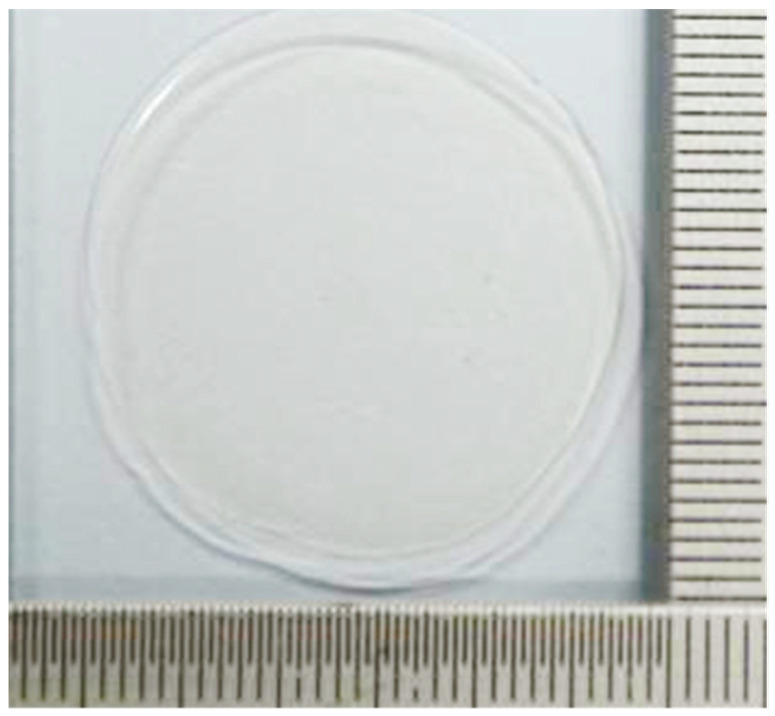
Macroscopic appearance of the free-standing film prepared from the optimal formulation.

Based on the single-factor results, PVA-124 (A), glycerol (B), and anhydrous ethanol (C) were selected for orthogonal optimization (L_9_(3^3^) array). The results and range analysis (R values) are presented in Table 8. The optimal formulation was determined to be A_2_B_2_C_2_, corresponding to 6.0 g PVA-124, 1.5 g glycerol, and 6.0 mL anhydrous ethanol, which yielded the highest composite score. A photograph of the free-standing film prepared from this optimal formulation is provided in Figure 1.

### 2.3. Characterization of the Optimized PAP

#### 2.3.1. Macroscopic Film Mechanical and Rheological Properties

The mechanical integrity of the free-standing films is critical for their function as a wound dressing. As summarized in Table 9, the PAP film (containing PAG) exhibited a tensile strength of 14.38 ± 2.72 MPa and an elongation at break of 252.41 ± 94.41%. In comparison, the vehicle film (without PAG) showed lower values for both tensile strength (8.85 ± 0.30 MPa) and elongation at break (182.83 ± 32.91%). The displacement at maximum force followed a similar trend (42.20 ± 18.90 mm for PAP vs. 30.08 ± 9.58 mm for vehicle). These results indicate that the incorporation of PAG not only did not compromise the film’s mechanical properties but unexpectedly enhanced both its strength and extensibility, suggesting a potential interaction between PAG and the polymeric matrix that reinforces the film structure.

The ease of application is a key practical attribute. Rheological analysis (Table 10) revealed that both the PAP and vehicle solutions had comparable viscosities at the tested shear condition (100 rpm), with values of 14.38 ± 2.72 mPa·s and 8.85 ± 0.30 mPa·s, respectively. This low viscosity range confirms the formulations exhibit shear-thinning behavior (as indicated by the low viscosity at this moderate shear rate relative to a likely higher zero-shear viscosity), which is essential for easy spreading upon application while maintaining stability on storage. The similarity in viscosity between the two groups suggests that PAG’s incorporation does not adversely affect the processing and application characteristics of the base formulation.

#### 2.3.2. Physical Stability

The optimized PAP formulation passed accelerated stability tests. No phase separation, precipitation, or significant color changes were observed after thermal stress (55 °C, 2 h), freeze–thaw cycling (−18 °C, 72 h), or high-speed centrifugation.

### 2.4. The PAG-Based Film-Forming Agent Accelerates Wound Closure and Demonstrates Efficacy Superior to the Vehicle Control

**Figure 2:**
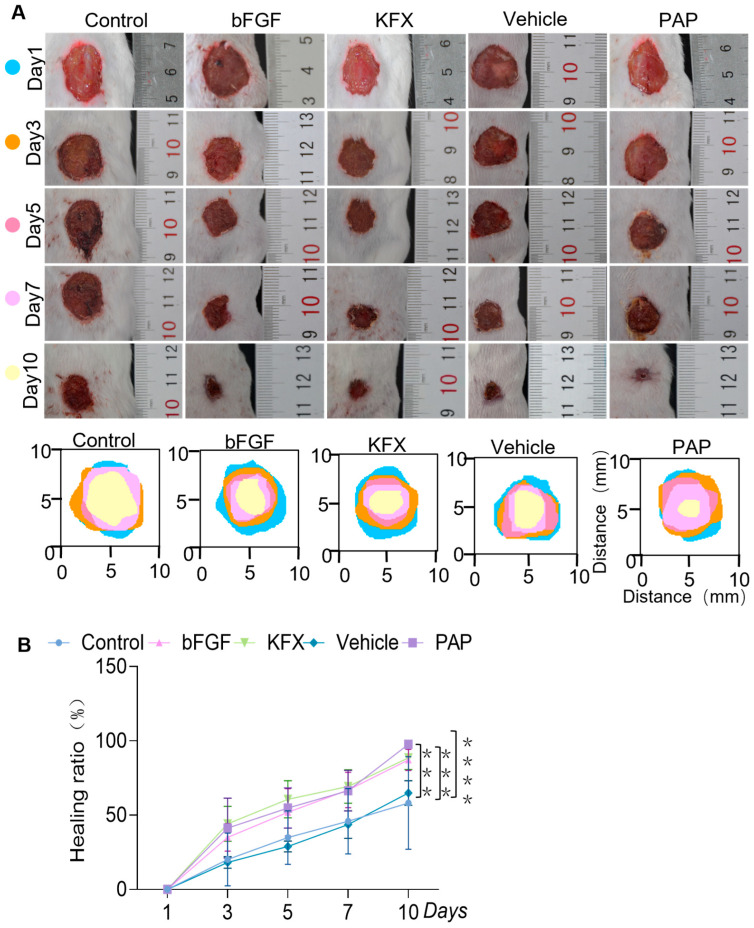
Effect of PAG coating agent on wound healing in mice. (**A**) Representative gross photographs of wounds treated with saline (Control), Vehicle (without PAG), bFGF, KFX, PAP at different time points. (**B**) Wound healing ratios of different treatment regimens at various time points. Data are presented as means ± standard deviation (*** p < 0.001, *n* ≥ 3, **** p < 0.0001, *n* ≥ 3).

All five experimental groups completed the study. Representative wound images and quantitative healing rates are shown in Figure 2. On day 3, wound edge contraction was initiated in the PAP and KFX groups, with more pronounced effects than in the Saline group. By day 7, the bFGF group showed intact scabs, while the PAP group exhibited only slight edge lifting. Critically, the Vehicle control group showed no significant improvement over the Saline group in terms of scab condition or closure rate at all time points, confirming that the observed pro-healing activity was attributable to PAG and not the film-forming matrix alone.

On day 10, the PAP group achieved near-complete healing, which was significantly higher than that of the Saline group and the Vehicle control group, and comparable to the bFGF and KFX groups (Figure 2A,B). No sex-based differences in healing progression were observed.

### 2.5. Histological Analysis Confirms Enhanced Tissue Regeneration and Collagen Deposition

**Figure 3:**
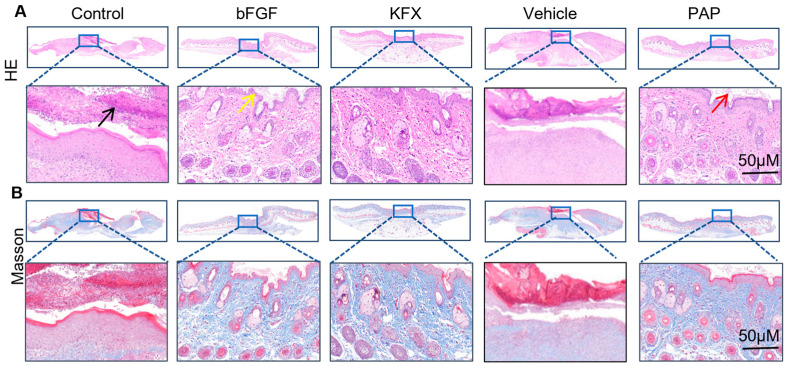
Histopathological analysis. (**A**) H&E staining and (**B**) Masson staining of the wound tissue on day 10 (Scale bar: 50 μm).

H&E and Masson’s trichrome staining of day-10 wound tissues provided mechanistic insights (Figure 3). Compared to the Saline and Vehicle groups, which displayed disorganized cell architecture, inflammatory infiltration, and disrupted collagen fibers (black arrow), the PAP group exhibited well-aligned cells, reduced inflammation, and densely organized collagen bundles (yellow arrow). Epidermal regeneration was markedly enhanced, with a thickened epidermis and closely packed keratinocytes (red arrow). The collagen deposition and organization in the PAP group were superior to those in the Saline group and comparable to the bFGF and KFX groups, indicating promotion of structured tissue remodeling.

### 2.6. PAP Exhibits Potent Free Radical Scavenging Activity In Vitro

**Figure 4:**
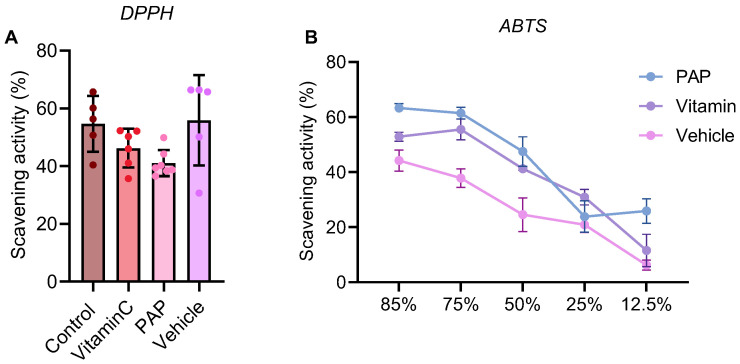
Evaluation of antioxidant activity. (**A**) DPPH radical scavenging capacity of PAP. (**B**) The antioxidant capacities of PAP via ABTS reduction (*n* = 3 independent experiments, data are presented as mean + S.D.).

PAP demonstrated potent free radical scavenging capacity (Figure 4). In the DPPH assay, its activity was comparable to that of vitamin C at equivalent concentrations. Similarly, in the ABTS assay, PAP exhibited strong radical cation scavenging activity, confirming its significant antioxidant potential.

### 2.7. PAP Shows Selective Antibacterial Activity, with Potent Inhibition Against S. aureus

**Figure 5:**
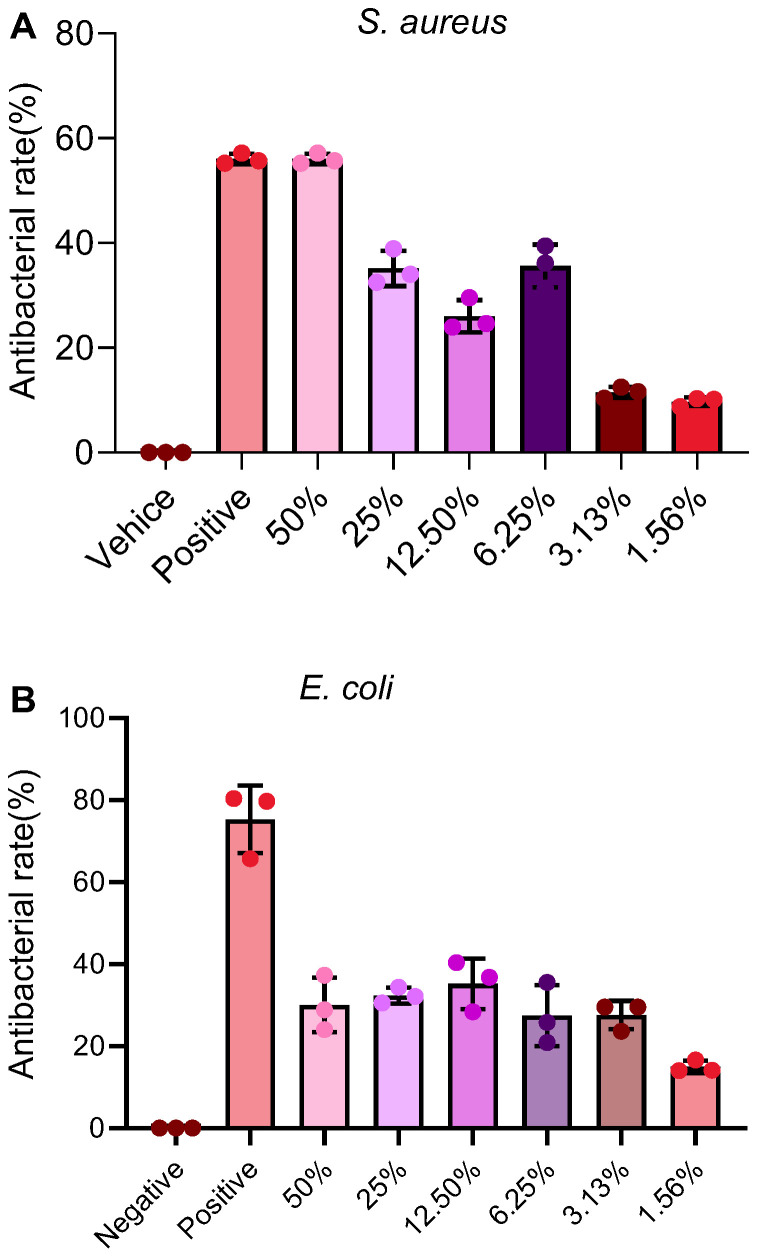
Bacteriostatic effects of PAP. Bacteriostatic effects of PAP on *S. aureus* (**A**), *E. coli* (**B**).

PAP showed a concentration-dependent inhibitory effect against *S. aureus*, with an inhibition rate of 56.02 ± 0.83% at the highest concentration tested (50% *v*/*v*) (Figure 5A). In contrast, its activity against *E. coli* was more limited, with a maximum inhibition of 32.34 ± 1.59% at the concentration of 25% (Figure 5B), clarifying the earlier descriptive discrepancy. This suggests a selective antibacterial profile, with greater potency against Gram-positive *S. aureus*.

## 3. Discussion

This study successfully developed and characterized a novel multifunctional PAP based on PAG. Moving beyond conventional liquid or semi-solid dosage forms, this optimized system—achieved through single-factor and orthogonal experimental design—demonstrates excellent film-forming properties, suitable mechanical strength, and desirable pseudoplastic rheology for easy application. More importantly, it integrates the intrinsic pro-healing, antioxidant, and antimicrobial activities of PAG into a patient-friendly topical film, presenting a comprehensive strategy for wound management.

The GC–MS analysis revealed PAG’s complex chemical profile, rich in heterocyclic compounds, alkanolamines, hydrocarbons, and terpenoids, including sterol-like analogs such as levo-borneol and β-pinene. This composition aligns with reports attributing the bioactivity of *P. americana* extracts to a synergy of components rather than a single molecule [26]. We hypothesize that the wound-healing efficacy arises from this synergy: sterols and terpenoids may modulate inflammatory pathways and promote fibroblast proliferation [27], while fatty acid derivatives could enhance membrane fluidity and tissue repair processes [2]. The reproducibility of such a natural product may be a legitimate concern. While absolute concentrations of specific compounds may vary with insect diet, age, and habitat—a common challenge in botanicals and insect-derived medicines—the identified overarching classes of bioactive compounds (e.g., sterols, terpenoids) are characteristic of the species. Standardization of the extraction protocol, as employed here, ensures a consistent fingerprint of these major bioactive classes, which is crucial for reproducible functional outcomes [28].

The selection of PVA-124 as the primary film-forming polymer was based on its proven safety, excellent film-forming ability, mechanical robustness, and high compatibility with other excipients [29]. While natural polymers like chitosan possess inherent biological activity, PVA offers superior batch-to-batch consistency, tunable mechanical properties, and clarity—critical for a film intended as a wound coating. The addition of PVP enhanced film toughness and adhesion, while glycerol provided essential plasticity. The shear-thinning behavior confirmed by rheology is a key asset; it ensures the formulation is viscous enough to stay in place upon application yet spreads easily under shear, significantly improving usability over conventional messy ointments or solutions.

A critical advancement in this study was the inclusion of a vehicle control group. Its performance in both mechanical testing and the in vivo wound model was pivotal. The vehicle film exhibited mechanical properties nearly identical to PAP, confirming that PAG does not weaken the film matrix. In vivo, the vehicle group’s healing trajectory was statistically indistinguishable from the saline control, providing direct and compelling evidence that the significant wound closure, reduced inflammation, and enhanced collagen remodeling observed in the PAP group are unequivocally attributable to the bioactive PAG fraction, not the polymeric carrier itself.

Chronic wounds are often trapped in a pathological cycle of persistent infection, excessive oxidative stress, and impaired cellular function [30,31]. PAP addresses these multiple barriers. Its antibacterial activity, particularly against *S. aureus*, is crucial as this pathogen is a common colonizer of skin wounds and a driver of biofilm formation [32,33]. The more limited activity against *E. coli* suggests a spectrum of action that may be shaped by the lipid-rich composition of PAG, often more effective against Gram-positive bacteria [33]. Concurrently, the potent free radical scavenging activity of PAP, demonstrated in both DPPH and ABTS assays, can neutralize excess ROS at the wound site. By mitigating oxidative damage, PAP likely protects fibroblasts and keratinocytes, creating a microenvironment more conducive to proliferation and migration [34].

These in vitro activities translate directly to the observed in vivo efficacy. The accelerated wound closure, rapid resolution of inflammation, and superior collagen deposition and organization in the PAP-treated group depict a coordinated healing process. The bioactive components in PAG appear to not only control potential infection and oxidative damage but also directly stimulate key reparative cellular activities, leading to higher-quality tissue regeneration comparable to established treatments like bFGF and KFX.

Despite these promising results, our study also has some limitations. First, while we identified the major chemical classes in PAG, the precise molecular entities most responsible for its pro-healing effects and their exact molecular targets (e.g., TGF-β/Smad or Wnt/β-catenin pathways) remain to be elucidated. Second, the murine full-thickness excision model represents an acute wound model in a healthy animal. The efficacy of PAP should be validated in models that better mimic clinically challenging chronic wounds, such as diabetic or ischemic ulcers, where the healing cascade is impaired. Third, detailed long-term stability studies, scale-up production parameters, and comprehensive safety assessments including sensitization potential are necessary before clinical translation.

## 4. Materials and Methods

### 4.1. Materials

The following instruments were employed in this study: Electronic Balance (EX224, Ohaus Instruments, Parsippany, NJ, USA); Digital Display Temperature-Controlled Magnetic Stirrer (RET B S025, IKA, Staufen, Germany); Blast Drying Oven (DHG-9240A, Shanghai Yiheng Scientific Instrument, Shanghai, China); Electric thermostatic water pot (HWS-24, Shanghai Yiheng Scientific Instrument, China); Tensile Strength Tester (HT-LL-969, Dongguan Huitai Machinery Co., Ltd., Dongguan, China); Universal Testing Machine (TM2101-T7-01, Dongguan Huitai Machinery Co., Ltd., China) for mechanical testing; High-Speed Refrigerated Centrifuge (HC-3018R, Anhui Zhongke Zhongjia Scientific Instrument, Hefei, China); Rotational rheometer (DHR-3, TA Instruments, New Castle, DE, USA) for viscosity measurements; GC-MS/MS (8890-7000E, Agilent, Santa Clara, CA, USA), chromatographic column (DB-5MS (30 m × 0.25 mm × 0.25 μm, Agilent, USA), solid-phase microextraction (CTC Analytics AG, Zwingen, Switzerland), and DVB/CWR/PDMS SPME Arrow fiber (120 µm film thickness, CTC Analytics AG, Switzerland), Viscosimeter (RVDV-1, Shanghai Fangrui Instrument Co., Ltd., Shanghai, China).

### 4.2. Drugs and Reagents

The *P. americana* L. dried whole insects were provided by Dr. Yunchuan Yang from the Institute of Insect Biomedicine, Dali University (batch number 20210513). For in vivo studies, the following were used: bovine basic fibroblast growth factor (03230504, Zhuhai Yisheng Biopharmaceutical, Zhuhai, China); Kangfuxin solution (230901, Sichuan Good Doctor Panxi Pharmaceutical, Xichang, China); isoflurane for animal anesthesia (R510-22-10, Shenzhen Ruiwode Life Science, Shenzhen, China). Film-forming matrix components and chemicals included: Polyvinyl alcohol 124 ((PVA-124), 151024, Xilong Chemical, Shantou, China); Polyvinyl pyrrolidone (PVP; 160310, Xilong Chemical); Propylene glycol (C1806012, Aladdin Biotechnology, Shanghai, China); Anhydrous ethanol (20231212, Shandian Pharmaceutical Co., Ltd. (Kunming, China), Yanglin Industrial Development Zone, Yunnan); Tween-80 (20221012, Sinopharm Chemical Reagent, Shanghai, China); Azone (161201, Hubei Kejie Pharmaceutical, Tianmen, China). Reagents for bioactivity assays were: Chloramphenicol (GC301018, Servicebio, Wuhan, China); Ampicillin (G4018, Servicebio); ABTS (37615, ThermoFisher, Waltham, MA, USA); DPPH ( T11093, TargetMol, Wellesley Hills, MA, USA); Hexyl hydride (1730733, Merck, Darmstadt, Germany). All other chemicals were of analytical or chromatographic grade.

### 4.3. Preparation of PAG

**Figure 6:**
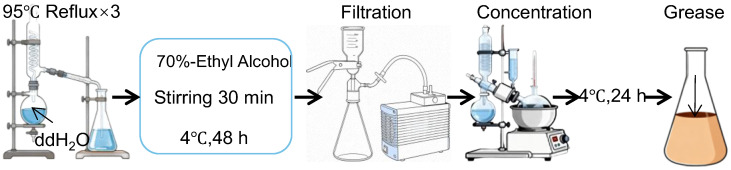
Procedures of the extraction and purification of PAG.

PAG was extracted as previously described with minor modifications [35]. Briefly, dried whole insects were mechanically crushed (Figure 6). Specifically, the dried whole insects were pulverized and subjected to triple reflux extraction with 10 volumes of purified water at 95 °C (2 h per cycle). The combined extracts were added to the concentrate to a final concentration of 70% (*v*/*v*). The mixture was stirred vigorously for 30 min and then stored at 4 °C for 48 h to promote phase separation. After filtration, the filtrate was concentrated again to remove the ethanol completely. The final concentrate was allowed to stand at 4 °C for 24 h, and the upper grease layer (PAG) was collected for subsequent use.

### 4.4. GC-MS Analysis

#### 4.4.1. Sample Preparation for Volatile Compound Analysis

The chemical profile of PAG was characterized by gas chromatography-mass spectrometry (GC-MS). For the analysis of volatile organic compounds (VOCs), samples were prepared using headspace solid-phase microextraction (HS-SPME). In detail, PAG (0.2 g) was accurately weighed and immediately transferred to a 20 mL head-space vial. To quench any enzymatic activity, NaCl (0.2 g) was added to the vial. The vial was sealed with a crimp-top cap equipped with a polytetrafluoroethylene (PTFE)/silicone septum. Prior to SPME extraction, the vial was equilibrated at 60 °C for 5 min in a heating block. A DVB/CWR/PDMS SPME Arrow fiber (120 µm film thickness) was then exposed to the headspace of the sample for 15 min at 60 °C to adsorb the VOCs.

#### 4.4.2. GC-MS Conditions

Following sampling, SPME Arrow fiber was thermally desorbed in the GC injection port at 250 °C for 5 min. Separation and detection were performed using an Agilent 8890 GC system coupled with a 7000E mass spectrometer. Analytes were separated on a DB-5MS capillary column. High-purity helium was used as the carrier gas at a constant flow rate of 1.2 mL/min. The GC oven temperature program was initiated at 40 °C (held for 3.5 min), increased to 100 °C at a rate of 10 °C/min, then to 180 °C at 7 °C/min, and finally to 280 °C at 25 °C/min (hold for 5 min). The injector temperature was maintained at 250 °C. Mass spectrometry detection was operated in electron impact (EI) mode at 70 eV. The temperatures of the ion source and transfer line were set at 150, 230 and 280 °C, respectively. Mass spectra were recorded in full-scan mode (m/z range 50–550) for compound identification. Tentative identification of the detected compounds was based on comparison with the standard mass spectra in the NIST 2017 library (https://nistmass.diabloanalytical.com). The primary chemical categories of the identified components were assigned with reference to authoritative databases, including PubChem and ClassyFire.

### 4.5. Preparation of Film-Forming Agent

The *Periplaneta* americana grease-based film-forming agent (PAP) was prepared using a two-phase mixing method.

Phase A: PVA-124 (6 g) was swollen in purified water for 24 h, and then completely dissolved in a 90 °C water bath. PVP (0.6 g) was added to the hot PVA solution with stirring until a clear solution was obtained.

Phase B: PAG (0.4 g), glycerol (1.5 g), and Tween-80 (0.1 g) were mixed using a thermostatic magnetic stirrer to form a homogeneous pre-emulsion. Anhydrous ethanol (6.0 mL) was then incorporated under continuous stirring. After both phases cooled to room temperature (25 ± 2 °C), Phase A was slowly added to Phase B under constant stirring. The mixture was stirred for an additional 10 min, followed by the addition of Azone (0.1%, *v*/*v*). The formulation was left to stand to allow complete dissipation of entrapped air bubbles, and the final volume was adjusted to 100 mL with purified water. A vehicle control formulation, identical in composition but without PAG, was prepared in parallel for comparative studies.

### 4.6. Evaluation of Film-Forming Properties

#### 4.6.1. Preliminary Evaluation Parameters

The film-forming characteristics were assessed based on five parameters: film formation time, film integrity, uniformity, bubble formation, and flexibility. These assessments were carried out via a tensile displacement method using dried threads from the solution, as a preliminary screening tool. Specific protocols remained as follows:

##### Film-Formation Characteristics

A defined volume (approximately 100 µL) of the film-forming solution was uniformly applied to the inner forearm of a volunteer. The time required for the solution to dry and form a continuous, adherent film was recorded as the film-formation time. The integrity of the resulting film (e.g., continuity, adhesiveness) and the presence of macroscopically visible bubbles were assessed visually.

##### Preliminary Assessment of Mechanical Properties

As an initial screening method [36] for flexibility, the solution was spread evenly onto a glass slide (2 cm × 2 cm) and dried in an oven at 35 °C until a solid film was obtained. The dried film was carefully peeled and manually rolled into a filament. The tensile displacement of the filament was measured using a tensile strength tester to obtain a relative indication of material toughness. It should be noted that this method served as a preliminary, qualitative assessment; comprehensive mechanical testing of standardized macroscopic films is reported in Section 4.6.3.

##### Physical Stability Evaluation

Samples of the liquid formulation were stored in sealed transparent vials at room temperature (25 ± 2 °C) for 72 h. They were periodically inspected for any signs of phase separation, precipitation, or changes in homogeneity.

#### 4.6.2. Comprehensive Mechanical Characterization of Macroscopic Films

To rigorously evaluate the mechanical properties relevant to the final dosage form, free-standing films were prepared and tested. The optimized PAP film-forming solution and its vehicle counterpart (without PAG) were cast onto leveled polytetrafluoroethylene (PTFE) plates. The films were dried in a controlled oven at 35 °C for 24 h to a final thickness of 0.10 ± 0.02 mm. The dried films were carefully peeled and cut into dumbbell-shaped specimens according to ASTM D882 standard dimensions (width: 10 mm, gauge length: 50 mm) [37]. Tensile testing was performed using a universal testing machine (e.g., Instron 5943) at a crosshead speed of 10 mm/min. Tensile strength, elongation at break, and displacement at maximum force were recorded directly from the force-displacement curves. Three replicate specimens were tested for each formulation.

#### 4.6.3. Rheological Characterization

The flow behavior of the liquid film-forming formulations was analyzed to assess their applicability. Steady-state viscosity measurements were conducted using a rotational rheometer (e.g., TA Instruments DHR-3) equipped with a cone-plate geometry (40 mm diameter, 1° cone angle). Measurements were performed at a constant temperature of 15.8 ± 0.1 °C. The apparent viscosity was recorded at a rotational speed of 100 rpm after reaching equilibrium. Three independent samples were measured for both the PAP and vehicle solutions.

### 4.7. Formulation Optimization via Single-Factor and Orthogonal Experiments

The formulation was optimized through sequential single-factor screening and orthogonal experimental design. Based on preliminary trials, six critical factors were identified for screening: the amounts of PVA-124, PVP, PAG, glycerol, Tween-80, and anhydrous ethanol (Table 11). For each factor, four concentration levels were tested. The comprehensive scoring system (Table 12) was applied to evaluate each trial batch. Factors exhibiting the most significant influence on the composite score (PVA-124, glycerol, and anhydrous ethanol) were selected for further optimization using a three-factor, three-level L_9_(3^3^) orthogonal array design. The experimental layout and results are presented in Table 13.

### 4.8. Stability Evaluation

The physical stability of the optimized PAP formulation was assessed under accelerated conditions. For thermal stability, triplicate samples were maintained at 55 ± 1 °C for 2 h in a water bath. Cold resistance was evaluated by storing samples at −18 °C for 72 h. Centrifugation stability was tested by subjecting samples to 6000× *g* for 10 min at 4 °C. All samples were visually inspected for phase separation, precipitation, or color change immediately after each test.

### 4.9. In Vitro Antioxidant Activity

The radical scavenging capacity of PAP was evaluated using DPPH and ABTS assays, with vitamin C as a positive control. For the DPPH assay, 100 µL of sample was mixed with 100 µL of a 0.1 mM DPPH ethanolic solution. After incubation in the dark at 37 °C for 30 min, absorbance was measured at 517 nm. The ABTS radical cation working solution was prepared by reacting 7 mM ABTS with 4.9 mM potassium persulfate for 15 h in the dark and then diluting to an absorbance of 0.70 ± 0.02 at 734 nm. For the assay, 0.2 mL of sample was mixed with 4.0 mL of ABTS working solution, reacted for 6 min, and absorbance was measured at 734 nm. Scavenging activity was calculated using standard formulas. All assays were performed in triplicate.

### 4.10. In Vitro Antibiotic Activity

The antibacterial activity was evaluated against Staphylococcus aureus (ATCC 25923) and Escherichia coli (ATCC 25922) using a broth microdilution method [38]. Bacterial suspensions were adjusted to approximately 1 × 10^3^ CFU/mL in fresh LB broth. Aliquots (100 μL) of bacterial suspension were added to wells containing 100 μL of serially diluted PAG or controls (chloramphenicol for *S. aureus*, ampicillin for *E. coli*). After incubation at 37 °C for 24 h, the bacterial growth was quantified by measuring the optical density at 600 nm (OD_600_) using a microplate reader. All experiments were conducted in at least three independent replicates. The bacteriostatic rate was determined using the formula: [(blank group value − experimental group value)/blank group value] × 100%.

### 4.11. In Vivo Wound Healing Study

#### 4.11.1. Animals and Ethical Statement

Male and female Kunming mice (20–25 g, 6–8 weeks old, SPF grade), were purchased from Hunan Slake Jingda Laboratory Animal Co., Ltd. (Changsha, China) (license No.: SYXK (Dian) 2018-0002). Animals were housed under standard conditions (22 ± 2 °C, 12 h light/dark cycle) with free access to food and water in the Laboratory Animal Center of Dali University. All experimental procedures were approved by the Animal Care and Use Committee of Dali University, China (Animal Ethics No.: #2024-PZ-157, approved on 10 September 2024).

#### 4.11.2. Grouping and Wound Model

After one week of acclimatization, mice were randomly divided into five experimental groups (*n* = 6 per group, 1:1 male-to-female ratio):

Saline group: Received topical application of sterile normal saline.

bFGF group: Received topical application of recombinant bovine basic fibroblast growth factor (bFGF, 35,000 IU/8 mL, 50 µL per wound) as a positive control.

KFX group: Received topical application of Kangfuxin solution.

PAP group: Received topical application of the optimized PAP.

Vehicle control group: Received topical application of the vehicle control (the film-forming agent without PAG).

Under isoflurane anesthesia, a full-thickness excisional wound (2 × 2 cm^2^) was created on the shaved dorsal skin. Wounds were cleaned with saline.

#### 4.11.3. Drug Treatment and Healing Assessment

Treatments (150 µL per wound) were applied topically three times daily for 10 consecutive days. Wound areas were photographed on days 1, 3, 5, 7, and 10 post-wounding. The wound area was measured using ImageJ software (v1.53, NIH, Bethesda, MD, USA), and the healing rate was calculated as: Healing rate (%) = [(Initial area − Area on Day n)/Initial area] × 100.

### 4.12. Histopathological Analysis

On day 10, the mice were deeply anesthetized with 1.5% isoflurane. Wound tissues were harvested and fixed in 4% paraformaldehyde (24 h at 4 °C), embedded in paraffin, and sectioned (5 μm thickness). Sections were stained with Hematoxylin and eosin (H&E) for general morphology and Masson’s trichrome stain for collagen deposition, following standard protocols. Stained sections were examined under a light microscope.

### 4.13. Statistical Methods

Data are presented as mean ± standard deviation (SD). Statistical analysis was performed using GraphPad Prism 9.0 software. For multiple group comparisons, one-way analysis of variance (ANOVA) followed by LSD post hoc LSD testing. For time-course data (wound healing rate), repeated-measures ANOVA was applied. A *p*-value of less than 0.05 was considered statistically significant.

## 5. Conclusions

In conclusion, this study demonstrates the successful development of a stable, multifunctional film-forming agent derived from PAG. The optimized formulation, PAP, exhibits the following key attributes: (1) excellent film-forming properties, suitable mechanical strength, and user-friendly shear-thinning rheology; (2) significant in vitro antioxidant activity and selective antibacterial efficacy; and (3) marked in vivo wound-healing effects, including accelerated wound closure, reduced inflammation, and enhanced structured collagen deposition—effects that are specifically attributable to the PAG fraction. Collectively, PAP offers a novel, integrated topical strategy that simultaneously addresses infection, oxidative stress, and tissue regeneration, representing a promising approach for the management of complex wounds.

This work not only advances the application of a traditional medicinal resource through modern pharmaceutical formulation but also establishes a solid preclinical foundation for the further development of PAP as a candidate for advanced wound care. Future studies will focus on elucidating its molecular mechanisms, evaluating its efficacy in models of chronic wounds, and conducting the necessary translational research.

## Figures and Tables

**Table 1:** Chemical composition and relative content of major compounds identified in PAG by GC-MS analysis.

	Chemical Components	Formula	Categories	Relative Content
1	2,3,5-trimethylpyrrole	C_7_H_11_N	Heterocyclic compound	9.774390
2	2-Ethyl-3,5-dimethylpyrazine	C_8_H_12_N_2_	Heterocyclic compound	7.620579
3	O-pentyl-Hydroxylamine	C_5_H_13_NO	Alcohol, amine compounds	7.211223
4	3,5-dimethyl-Heptane	C_9_H_20_	Hydrocarbon	5.006425
5	3-ethyl-Heptane	C_9_H_20_	Hydrocarbon	5.003612
6	4-Methylthiazole	C_4_H_5_NS	Heterocyclic	4.854652
7	2,3-Dimethyl-4-penten-2-ol	C_7_H_14_O	Alcohol, amine compounds	2.943017
8	2-methyl-Cyclopentanol	C_6_H_12_O	Alcohol, amine compounds	2.757836
9	P-Cresol	C_7_H_8_O	Monoaromatic Hydrocarbons	2.221379
10	3-methyl-2-Heptene	C_8_H_16_	Hydrocarbons	1.738263
11	1-Hepten-4-ol	C_7_H_14_O	Alcohol, amine compounds	1.542133
12	2-ethyl-6-methyl-Pyridine	C_8_H_11_N	Heterocyclic	1.259697
13	4-methyl-Octane	C_9_H_20_	Hydrocarbons	1.086163
14	Fomepizole	C_4_H_6_N_2_	Heterocyclic	1.070180
15	Sterol analogues	——	Terpene	1.053265

**Table 2:** Effect of PVA-124 concentration on the comprehensive scores of film-forming properties.

Evaluation Indicators	PVA-124			
	4 g	6 g	8 g	10 g
Film formation time	1.5	1.4	1.4	1.0
Film-forming	1.7	1.7	1.8	1.6
Homogeneity	1.8	1.7	1.8	1.7
With or without air bubbles	1.5	1.8	1.7	1.6
Flexibility	1.6	1.6	1.3	1.4
Overall rating	8.1	8.2	8.0	7.3

**Table 3:** Effect of PVP concentration on the comprehensive scores of film-forming properties.

Evaluation Indicators	PVP			
	0.2 g	0.4 g	0.6 g	0.8 g
Film formation time	1.0	1.4	1.3	1.0
Film-forming	1.2	1.6	1.7	1.3
Homogeneity	1.6	1.7	1.7	1.7
With or without air bubbles	1.0	1.6	1.7	1.3
Flexibility	1.0	1.6	1.7	1.6
Overall rating	5.8	7.9	8.1	6.9

**Table 4:** Effect of glycerol concentration on the comprehensive scores of film-forming properties.

Evaluation Indicators	Glycerine			
	0.5 g	1.0 g	1.5 g	2.0 g
Film formation time	1.2	1.4	1.4	1.4
Film-forming	1.7	1.8	1.5	1.4
Homogeneity	1.7	1.7	1.7	1.7
With or without air bubbles	1.6	1.7	1.5	1.4
Flexibility	1.0	1.4	1.8	1.5
Overall rating	7.2	8.0	7.9	7.4

**Table 5:** Effect of anhydrous ethanol volume on the comprehensive scores of film-forming properties.

Evaluation Indicators	Anhydrous Ethanol			
	2 mL	4 mL	6 mL	8 mL
Film formation time	1.3	1.3	1.8	1.6
Film-forming	1.3	1.7	1.5	1.5
Homogeneity	1.7	1.8	1.8	1.8
With or without air bubbles	1.3	1.5	1.5	1.4
Flexibility	1.8	1.5	1.5	1.4
Overall rating	7.4	7.8	8.1	7.7

**Table 6:** Effect of Tween-80 concentration on the comprehensive scores of film-forming properties.

Evaluation Indicators	Tween-80			
	0.05 g	0.1 g	0.15 g	0.2 g
Film formation time	1.5	1.0	1.0	1.4
Film-forming	1.7	1.8	1.8	1.5
Homogeneity	1.8	1.8	1.8	1.7
With or without air bubbles	1.6	1.6	1.8	1.4
Flexibility	1.3	1.6	1.5	1.6
Overall rating	7.9	7.8	7.9	7.6

**Table 7:** Effect of PAG loading on the comprehensive scores of film-forming properties.

Evaluation Indicators	PAG			
	0.2 g	0.3 g	0.4 g	0.5 g
Film formation time	1.3	1.7	1.7	1.2
Film-forming	1.6	1.7	1.7	1.7
Homogeneity	1.8	1.8	1.7	1.5
With or without air bubbles	1.7	1.7	1.6	1.7
Flexibility	1.7	1.2	1.5	1.5
Overall rating	8.1	8.1	8.2	7.6

**Table 8:** Design and results of the L_9_(3^3^) orthogonal experiment for optimizing the formulation of the film-forming agent.

Groups	A	B	C	D (Blank Group)	Y (Composite Score)
1	1 (4.0 g)	1 (1.0 g)	1 (4.0 mL)	1	8.1
2	1 (4.0 g)	2 (1.5 g)	2 (5.0 mL)	2	6.7
3	1 (4.0 g)	3 (2.0 g)	3 (6.0 mL)	2	5.1
4	2 (5.0 g)	1 (1.0 g)	2 (5.0 mL)	1	6.6
5	2 (5.0 g)	2 (1.5 g)	3 (6.0 mL)	3	5.4
6	2 (5.0 g)	3 (2.0 g)	1 (4.0 mL)	3	2.5
7	3 (6.0 g)	1 (1.0 g)	3 (6.0 mL)	1	4.4
8	3 (6.0 g)	2 (1.5 g)	1 (4.0 mL)	3	5.7
9	3 (6.0 g)	3 (2.0 g)	2 (5.0 mL)	2	4.8
Mean value 1	3.9	5.1	5.5	6.4	
Mean value 2	5.7	6.1	5.7	5.5	
Average value 3	6.8	5.3	5.2	4.5	
R	1.8	0.8	0.5	0.9	

**Table 9:** Mechanical properties of the macroscopic PAP and vehicle control film.

Group	Tensile Strength (MPa)	Elongation at Break (%)	Displacement at Maximum Force (mm)
PAP (containing PAG)	14.38 ± 2.72	252.41 ± 94.41	42.20 ± 18.90
Vehicle (without PAG)	8.85 ± 0.30	182.83 ± 32.91	30.08 ± 9.58

Data are presented as mean ± standard deviation (SD, *n* = 3).

**Table 10:** Rheological parameters of the PAP film-forming solution.

Group	Viscosity (mPa·s)	Rotational Speed (rpm)	Temperature (°C)
PAP (containing PAG)	14.38 ± 2.72	100	15.7
Vehicle (without PAG)	8.85 ± 0.30	100	15.8

Data are presented as mean ± standard deviation (SD, *n* = 3).

**Table 11:** Factors and concentration levels investigated in the single-factor experiments.

Factors	Level 1	Level 2	Level 3	Level 4
PVA-124 (g)	4.0	6.0	8.0	10.0
PVP (g)	0.2	0.4	0.6	0.8
PAG (g)	0.2	0.3	0.4	0.5
Glycerol (g)	0.5	1.0	1.5	2.0
Tween-80 (g)	0.05	0.10	0.15	0.20
Ethanol (mL)	4.0	6.0	8.0	10.0

**Table 12:** Scoring criteria and evaluation parameters for the film-forming performance.

Parameter	Evaluation Method	Scoring Scale (Max = 2.0)
Formation time	Average of triplicate measurements (min)	≤6: 1.5–2.0; 6–8: 1.0–1.5; ≥8: 0–1.0
Film integrity	Visual inspection after lifting	Complete: 2.0; Partial: 1.4–1.8; Poor: 0–1.3
Bubble formation	Microscopic examination	None: 2.0; Minor: 1.6–1.8; Significant: 0–1.5
Tensile strength	Tensile displacement (mm, mean of 3)	15.0–32.0: 1.2–2.0; <15: 0; ≥32: 0.5–1.1
Homogeneity	72 h stability test	No precipitation: 1.6–2.0; Precipitation: 1.0–1.5; Phase separation: 0

**Table 13:** Factors and levels design for the orthogonal experiment (L_9_(3^3^) array).

level of Achievement, etc.	PVA-124 (A)	Glycerine (B)	Ethanol (C)
1	4.0 g	1.0 g	4.0 mL
2	5.0 g	1.5 g	5.0 mL
3	6.0 g	2.0 g	6.0 mL

## Data Availability

The original contributions presented in this study are included in the article. Further inquiries can be directed to the corresponding authors.
